# Gr/3D–ZnO Nanocomposites as Humidity Sensors with Enhanced Sensing Response

**DOI:** 10.3390/polym13101623

**Published:** 2021-05-17

**Authors:** Wang-De Lin, You-Chen Lin, Ren-Jang Wu, Murthy Chavali

**Affiliations:** 1 Department of Center for General Education, St. Mary’s Junior College of Medicine, Nursing and Management, Yilan 26647, Taiwan; 2 Department of Applied Chemistry, Providence University, Taichung 43301, Taiwan; lisalisa3062988@gmail.com (Y.-C.L.); wurenjang@gmail.com (R.-J.W.); 3 NTRC-MCETRC and Aarshanano Composite Technologies Pvt. Ltd., Guntur District, Medikonduru 522201, India; ChavaliM@gmail.com

**Keywords:** graphene, ZnO, humidity sensor, nanocomposite

## Abstract

This work introduces a novel humidity sensor based on a nanocomposite material comprising graphene decorated with three-dimensional flower-like structures of zinc oxide (Gr/3D–ZnO) fabricated via a hydrothermal method with various weight percentages of graphene. The surface structure and morphology of the Gr/3D–ZnO nanocomposite were analyzed using XRD, EDS, SEM, TEM, and Raman spectroscopy. The influence of humidity on the electrical properties of the nanocomposite was also investigated. Experiment results revealed that the nanocomposite with 70 wt% of graphene provided high sensitivity (S = 446) with rapid response times (120 s) and recovery times (160 s). These results demonstrate the excellent potential of the proposed Gr/3D–ZnO nanocomposite in monitoring atmospheric humidity. A discussion on the mechanism underlying the effects of humidity on the Gr/3D–ZnO nanocomposite is also provided.

## 1. Introduction

Atmospheric levels of water vapor (i.e., humidity) can profoundly affect electronic devices, meteorological phenomena, agriculture, manufacturing, and food storage [1]. Efforts to control humidity levels depend on accurate measurements [2]; therefore, researchers are constantly striving to develop low-cost humidity sensors with ever-higher sensitivity and accuracy and ever-faster response and recovery times [3].

Numerous types of humidity sensors have been developed using resistive [4,5] and capacitive devices [6,7], quartz crystal microbalances [8,9,10], optical devices [11,12], and even surface acoustic waves [13,14]. This very particular research has also led to the development of materials and nanostructures that are highly sensitive to humidity, including TiO_2_ nanopowders [15] and nanotubes [16], ZnO nanowires [17], WO_3_ nanosheets [18], Fe_2_O_3_ nanopowders [19], graphene layers [20], organic/inorganic polymers [21], ZrO_2_ nanorods [22], and NiO-SnO_2_ nanofibers [23]. Among the various metal oxides used for humidity sensing, n-type Zinc oxide (ZnO) semiconductors offer low cost, a high surface area ratio, high stability, and excellent chemical reactivity [24]. ZnO can be synthesized into various nanostructures, such as nanowires [25], nanorods [26], nanosheets [27], nanoparticles [28], nanofibers [29], hollow spheres [30], and nanoflowers [31]. Despite these successes, and because of the extremely exposed surface area, ZnO often suffers from self-aggregation, which may reduce the materials effective area to lower performance. Poor response in low humidity environments and large hysteresis limits the application of pure ZnO [32]. Nonetheless, sensing performance can be improved through the control of morphology and other modification methods, such as doping and the formation of compounds [33]. Graphene, a transparent single-layer two dimensional of sp^2^ bonded carbon atoms, has a large surface to volume ratio and excellent conductivity [34,35,36], which makes it very suitable for humidity sensing material. Humidity sensors based on graphene sensing layer have been developed by researchers and exhibit the advantage of high sensitivity and fast response recovery to different humidity [37,38,39,40,41]. The hydrophobic nature of pure graphene limits its applicability in humidity sensors; however, researchers have demonstrated the potential of decorating graphene with metal oxide nanoparticles to fabricate high-performance humidity sensors [42,43]. The incorporation of metal oxide within RGO sheets increases the number of active sites (e.g., vacancies and defects), which greatly enhances humidity sensing performance [44,45].

Our objective in this study was to fabricate a sensor based on a nanocomposite material comprising graphene decorated with three-dimensional flower-like structures of zinc oxide (Gr/3D–ZnO). The surface structure and morphology of the Gr/3D–ZnO nanocomposite were characterized using XRD, EDS, SEM, TEM, and Raman spectroscopy. We also investigated the influence of humidity on the electrical properties of the nanocomposite. The completed humidity sensor exhibited ultra-high sensitivity with fast recovery and response times over a broad range of relative humidity (RH) levels.

## 2. Experimental

### 2.1. Materials

Commercial graphene film of high purity (>99%) was purchased from UniRegion Bio-Techn (New Taipei City, Taiwan) and Sigma–Aldrich Co., Inc. (St. Louis, MO, USA). Zinc nitrate hexahydrate (Zn(NO_3_)_2_·6H_2_O, 98%), anhydrous alcohol (C_2_H_5_OH, 99.5%), L-histidine (C_6_H_9_N_3_O_2,_ ≥ 99% ), urea ((NH_2_)_2_CO, 99.5%), and polyvinyl alcohol (PVA, 99+% hydrolyzed) were purchased from Sigma-Aldrich Co., Inc.

### 2.2. Fabrication of Gr/3D–ZnO Nanocomposite Material

The 3D–ZnO structures prepared asper the methods outlined in [46]. Briefly, 100 mL of 0.1 M zinc nitrate hexahydrate (Zn(NO₃)₂.6H₂O) and 10 mL of 1 M urea were mixed for 5 min. To this solution was slowly added 20 mL of an aqueous solution containing 0.05 M of L-histidine while stirring roughly 15 min at room temperature (RT). The mixture was then transferred into a Teflon-lined autoclave for hydrothermal treatment at 120 °C for 4 h. The resulting precipitate was collected via centrifugation and washed using deionized water and ethanol before being dried under a vacuum at 80 °C. Finally, Gr/3D–ZnO was obtained via calcination at 400 °C for 2 h. Tests were performed on nanocomposite sensing materials fabricated using graphene in various ratios (10, 20, 30, 50, 70, and 80 wt%).

### 2.3. Characterization

The surface morphology and structure of the proposed Gr/3D–ZnO nanocomposite were examined using transmission electron microscopy and energy-dispersive X-ray spectroscopy (TEM/EDS; JEM-2100F). The crystal structure of Gr/3D–ZnO was characterized using an XRD-6000 Shimadzu X-ray diffractometer with Cu Kα radiation of 1.5405 Å at 40 kV and 30 mA between 10° and 80° (2θ) at intervals of 2°/min. Infrared spectra were measured using an Agilent Cary spectrophotometer. The characteristic Raman spectrum of the Gr/3D–ZnO nanocomposite was measured at RT under excitation from a laser source at a wavelength of 532 nm (Jobin Yvon iHR550, HORIBA).

### 2.4. Sensor Fabrication and Humidity Testing

A solution of 10% PVA was added to promote the binding of the composite. A pair of comb-like gold electrodes were applied to an alumina substrate via dip-coating (10 × 5 mm^2^), whereupon the chips were heated to 80 °C and held at that temperature for 0.5 h before undergoing calcination at 400 °C for 2 h.

**Figure 1:**
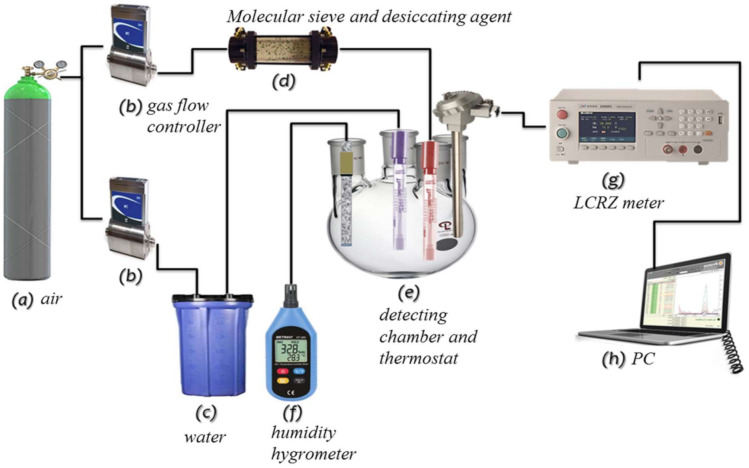
Experimental setup.

The experiment setup used to assess the performance of the humidity sensor is described in our previous work [42]. Briefly, the sensitivity of the device to humidity was measured in a dynamic flow system with the sensors held in an airtight glass chamber maintained at 25 °C ± 2 °C (Figure 1). Air was injected into the water to generate water vapour, which was subsequently pumped into the testing chamber. The specific RH was maintained at a given level for 15 min to enable the system to reach equilibrium. A thermo-hygrometer (Rotronic) with an accuracy of ±0.1% was connected to the testing chamber to obtain ground-truth measurements of the RH within. This commercial humidity sensor was calibrated to a standard concentration at Center of Measurement Standards, Industrial Technology Research Institute, Chutung, Hsinchu, Taiwan 31040. The sensing response (S) of the sensor to fluctuations in RH was defined as S = R_d_/R_h_, where R_d_ is the resistance of the sensor under dry conditions (12% RH) and R_h_ is the resistance under a specific level of humidity [43]. The humidity hysteresis properties were investigated by increasing the humidity from 12% to 90% and then decreasing it to 12% to facilitate the adsorption and desorption of water molecules.

Humidity hysteresis error (H) was calculated using the following equation:

H = Δf_max_/ffs × 100%,

where Δfmax indicates the maximum hysteresis error and ffs indicates the full-scale response output [47]. The response and recovery times were defined as the time required for the impedance of the sensor to change by a value equal to 90% of the total impedance. Recovery time was defined as the time required for the process to be reversed [48]. RH was adjusted dynamically between 12% and 90% by altering the air:water ratio fed into the chamber. The impedance response of the sensing material to ambient humidity was measured using a chemical impedance analyzer (Delta United, DU 6010) with an input voltage of 1 V operating at a frequency of 1 kHz.

## 3. Results and Discussion

### 3.1. Characterization of the Nanocomposite Structure

**Figure 2:**
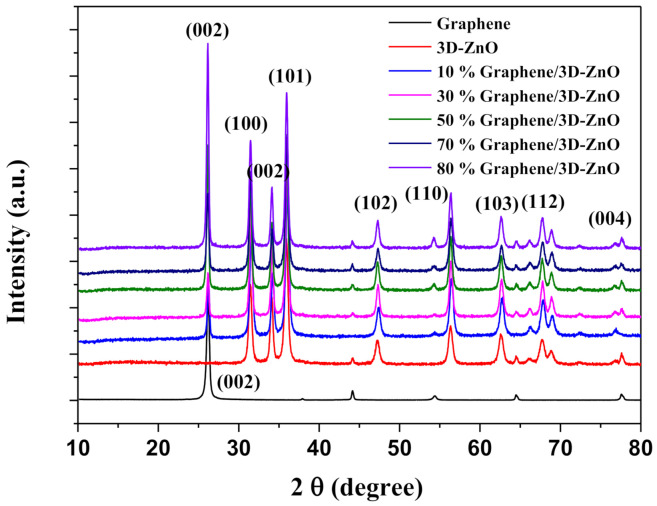
XRD analysis of Gr/3D–ZnO nanocomposite.

Figure 2 presents the XRD spectra of graphene, 3D–ZnO, and Gr/3D–ZnO with G concentrations of 10, 20, 30, 50, 70, and 80 wt%. As shown in Figure 2, XRD peaks indicative of graphene were observed at 26.42° [49]. The peaks at 31.6°, 34.2°, 36.2°, 47.5°, 56.4°, 62.7°, 67.7°, and 72.2° (2θ degrees), respectively corresponding to the (100), (002), (101), (102), (110), (103), (112) and (004) planes, similar to the typical hexagonal wurtzite structure of 3D–ZnO (JCPDS standard card No. 36-1451) [32]. Figure 2 present the spectra of five Gr/3D–ZnO samples, which revealed no new crystalline phases. Overall, these results demonstrate that the sensing material was a simple mixture.

**Figure 3:**
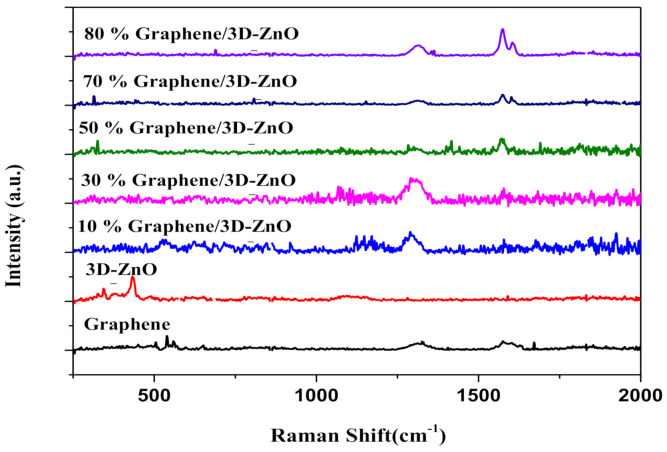
Raman spectra of Gr/3D–ZnO nanocomposite.

As shown in Figure 3, the crystalline structure of the samples was confirmed via Raman spectroscopy. Two prominent peaks indicative of defects in the D and G positions (at 1301 cm^−1^ and 1568 cm^−1^) were presumably due to in-plane bond stretching of sp^2^ hybridized carbon atoms and the corresponding combinational modes and overtones [50,51]. The peaks at 342 and 430 cm^−1^ are the vibrational modes of ZnO [52]. The Gr/3D–ZnO nanocomposite did not present any peaks indicative of ZnO.

**Figure 4:**
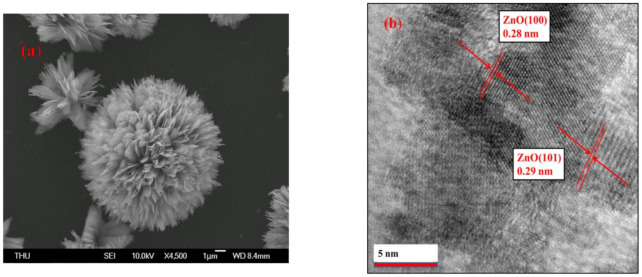

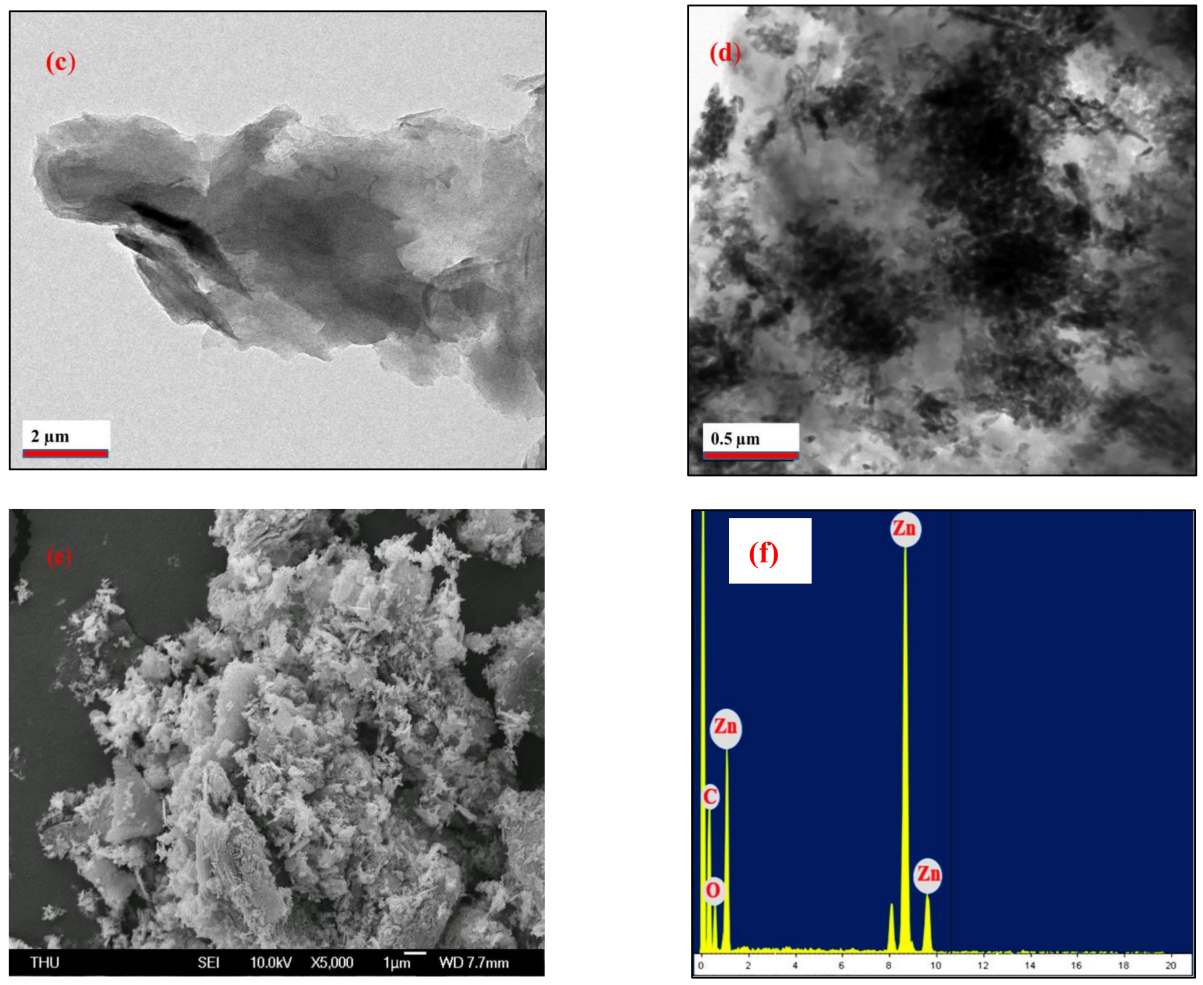
(**a**) SEM image of 3D–ZnO flower-like structure, (**b**) TEM image of 3D–ZnO, (**c**) TEM image of graphene sheets, (**d**) TEM image of 70 wt% Gr/3D–ZnO, (**e**) SEM image of 70 wt% Gr/3D–ZnO nanocomposite, (**f**) EDX spectrum of 70 wt% Gr/3D–ZnO nanocomposite.

Figure 4 presents the SEM and TEM results used to characterize the morphology of the 3D–ZnO, graphene, and 70 wt% Gr/3D–ZnO nanocomposite. The SEM image in Figure 4a illustrates the flower-like structure of 3D–ZnO. Figure 4b presents fringe spacing (0.28 nm and 0.29 nm) respectively indexed to the (100) and (101) planes of pure Wurtzite associated with the hexagonal structure of ZnO [53]. Figure 4c shows smooth graphene sheets stacked irregularly. Figure 4d,e reveals an abundance of 3D–ZnO aggregate on the surface of the 70 wt% Gr/3D–ZnO nanocomposite material. The EDS results in Figure 4f revealed peaks indicative of Zn, C, and O in the 70 wt% Gr/3D–ZnO nanocomposite. Overall, these results confirm the phase purity of the nanocomposite material.

### 3.2. Sensitivity to Humidity

**Figure 5:**
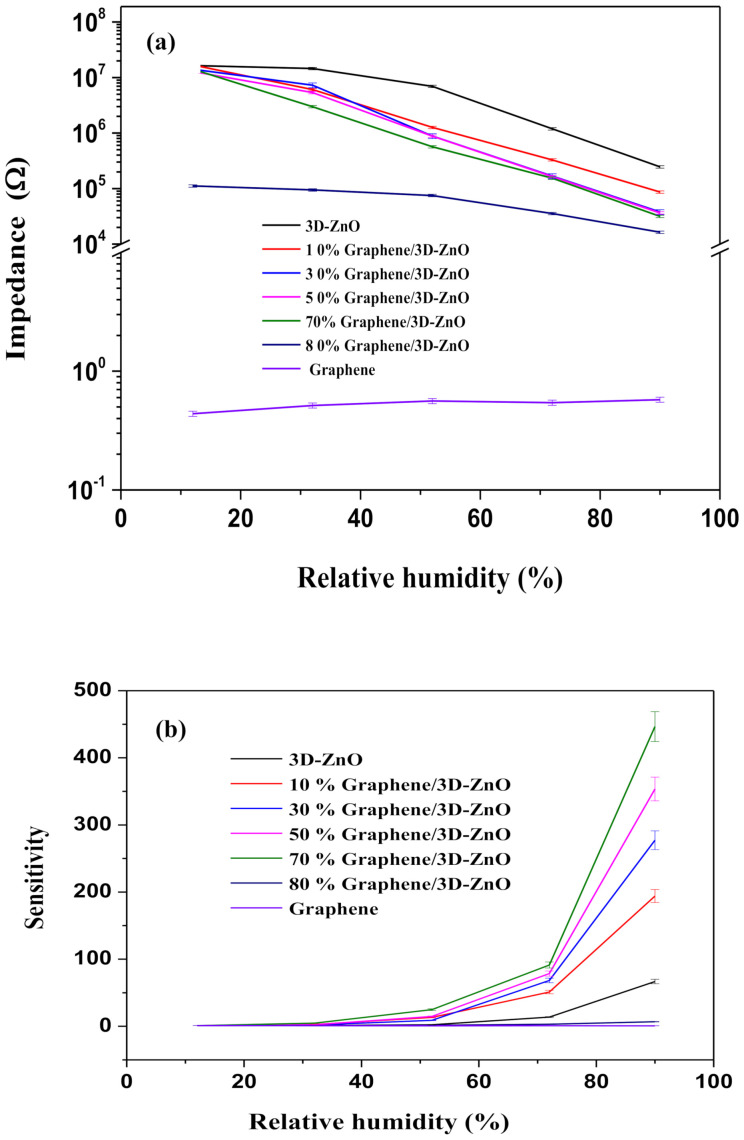
(**a**)Variation of impedance; (**b**) Variations in sensing response with the change in relative humidity (%) for different weight percentages of graphene in Gr/3D–ZnO nanocomposites.

We assessed the sensitivity of Gr/3D–ZnO nanocomposite materials (Gr concentrations of 10, 20, 30, 50, 70, and 80 wt%) to humidity based on impedance measurements obtained using AC voltage (1V) at a frequency of 1 kHz. As shown in Figure 5a, the impedance of the sensor material decreased exponentially with an increase in the RH. As shown in Figure 5b, samples of 70 wt% Gr/3D–ZnO exhibited the highest sensitivity (S = 446) across the range of humidity levels tested in this study (12–90%). We have selected 70 wt% Gr/3D–ZnO for all the subsequent analysis.

**Figure 6:**
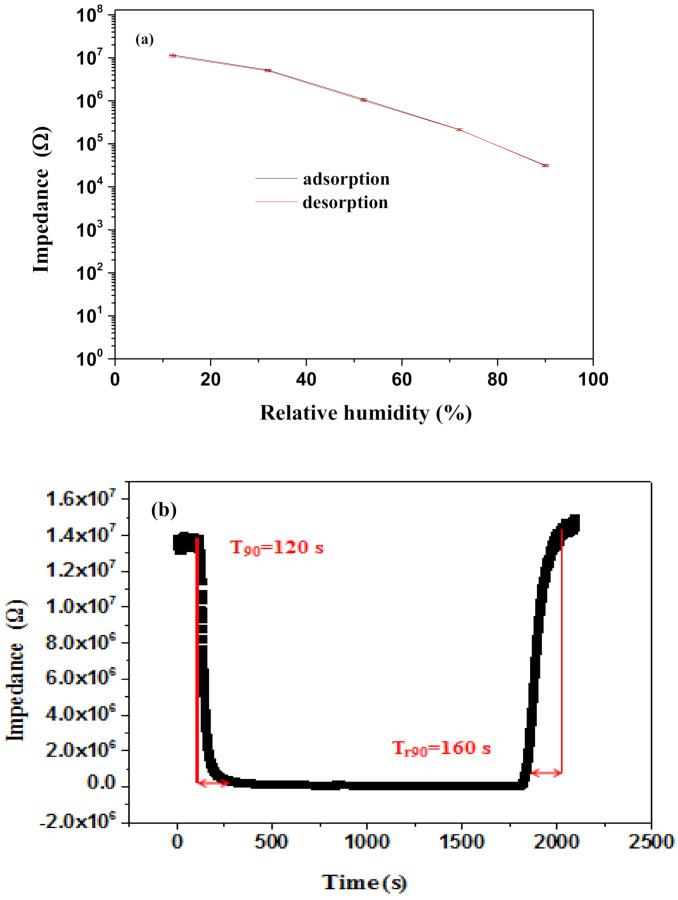
(**a**) Hysteresis characteristics (**b**) Response and recovery characteristics of 70 wt% Gr/3D–ZnO nanocomposites.

Humidity hysteresis is an important factor in evaluating the reliability of humidity sensors [47]. As shown in Figure 6a, the 70 wt% Gr/3D–ZnO nanocomposite exhibited excellent reversibility, with nearly overlapping adsorption and desorption curves. The maximum hysteresis (2.32%) occurred under RH of 90%.

Response and recovery times largely determine the practical applicability of humidity sensors. As shown in Figure 6b, the impedance of 70 wt% Gr/3D–ZnO nanocomposite decreased rapidly with an increase in RH until reaching a relatively stable value (a total of 120 s). The impedance then returned rapidly to the original value in a very short time when humid air was replaced with dry air (a total of 160 s). Table 1 compares the proposed 70 wt% Gr/3D–ZnO sensor with previous works in terms of sensitivity, response times, and recovery times [17,24,54,55,56,57]. Overall, the proposed Gr/3D–ZnO sensor performs well with all of the existing systems examined in this study.

**Figure 7:**
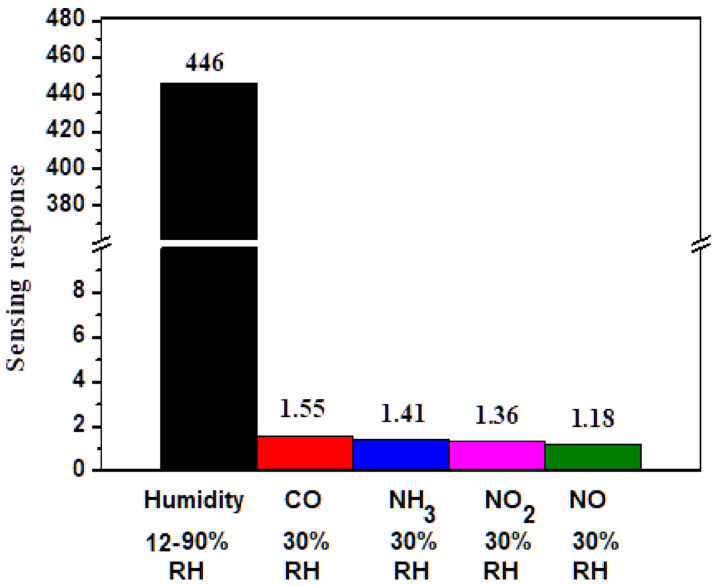
Selectivity for 70 wt% Gr/3D–ZnO nanocomposite towards humidity (12–90% RH), and gases like NO, NO_2_, NH_3_, CO (30% RH) at RT.

Selectivity is another crucial parameter in assessing the performance of humidity sensors. Figure 7 illustrates the selectivity of the 70 wt% Gr/3D–ZnO nanocomposite to an increase in humidity from 12% to 90% RH and various gases at concentrations of 1000 ppm under RT. The response of 70 wt% Gr/3D–ZnO nanocomposite to humidity was very high (R_12%_/R_90%_ = 446). The response to other gas species was as follows: CO (1.55), NH_3_ (1.41), NO_2_ (1.36), and NO (1.18). Overall, the Gr/3D–ZnO nanocomposite exhibited excellent selectivity for humidity.

**Figure 8:**
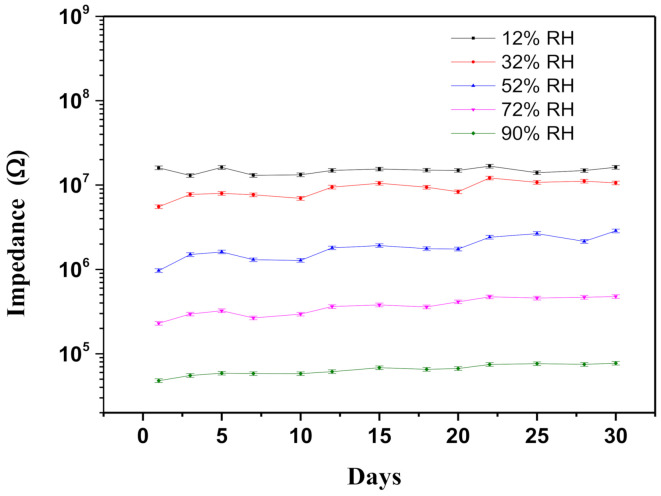
Long term stability of 70 wt% Gr/3D–ZnO nanocomposites at RT.

Figure 8 illustrates the stability of the prepared sensors as indicated by fluctuations in resistances under ambient RH of 12–90%. Only slight fluctuations in resistance were recorded over the 30-day testing period, regardless of the RH% environment.

### 3.3. Sensing Mechanism

**Figure 9:**
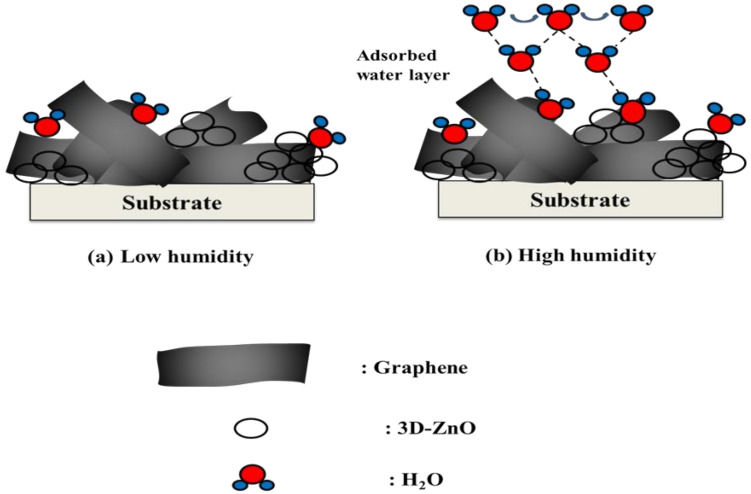
Scheme of humidity sensing mechanism (**a**) low humidity; (**b**) High humidity for Gr/3D–ZnO nanocomposite structure.

At low relative humidity (RH), the probability of contact between water molecules and Gr/3D–ZnO nanocomposite particles was low, so only the outer particles came into contact with the water molecules, as shown in Figure 9a. In this process, since water molecules could not form a continuous water layer, the transfer of H_2_O or H_3_O^+^ was on the discontinuous water layer was difficult [58]. Therefore, the Gr/3D–ZnO nanocomposite exhibited higher impedance at lower humidity. A relative high humidity (RH), one or several serial water layers were formed among the Gr/3D–ZnO nanocomposite particles, as presented in Figure 9b. The serial water layers accelerated the transfer of H_2_O or H_3_O^+^. Grotthuss and Casalbore-Miceli et al. outlined an ion transfer mechanism involving the transfer of H_2_O or H_3_O^+^ on serial water layers: H_2_O + H_3_O^+^ → H_3_O^+^ + H_2_O [59,60]. This implies that the free movement of conduction ions increased the electrical conductivity of the Gr/3D–ZnO nanocomposite, which made it highly sensitive to humidity with quick response and recovery times.

## 4. Conclusions

In this work we have succeeded in fabricating Gr/3D–ZnO nanocomposites via a hydrothermal process to create a novel low-cost humidity sensor based on Gr/3D–ZnO nanocomposite material. Experiment results revealed that the nanocomposite with 70 wt% graphene provided a high sensitivity (S = 446) with rapid response times (120 s) and recovery times (160 s). The highest humidity hysteresis error was only 2.32% over a wide range of RH values. These results demonstrate the excellent potential of the proposed Gr/3D–ZnO nanocomposite for the development of low-cost sensors to monitor atmospheric humidity.

## Figures and Tables

**Table 1:** Comparison of humidity sensing ability of modified ZnO-based sensors that were previously reported.

Sensing Material	Measurement Range (% RH.)	Response Time (s)	Recovery Time (s)	References
ZnO nanowires	11–97	35.3	32.6	[17]
PVDF/ZnO nanocomposites	5–95	30	50	[24]
ZnO nanosheets	12–96	600	3	[54]
Sn-doped ZnO nanorod	40–90	230	30	[55]
LiCl-doped ZnO electrospun nanofibers	11–95	3	6	[56]
ZnO colloid crystals	5–98	152	10	[57]
Gr/3D–ZnO nanocomposites	12–90	120	160	This work
